# Dr. Ignaz Phillip Semmelweis: The Unrecognized Pioneer of Aseptic Practices

**DOI:** 10.7759/cureus.68350

**Published:** 2024-08-31

**Authors:** Nistha R Dash, Gaurav Singh, Atish Mohapatra, Sharan S Keshetty

**Affiliations:** 1 Department of Community and Family Medicine, All India Institute of Medical Sciences, Raipur, Raipur, IND; 2 Department of Microbiology, All India Institute of Medical Sciences, Raipur, Raipur, IND; 3 Department of Pediatric Surgery, All India Institute of Medical Sciences, Raipur, Raipur, IND

**Keywords:** ignaz semmelweis (1818-1865), semmelweis reflex, puerperal fever, handwashing practices, medical pioneer, historical vignette

## Abstract

Modern medicine is well-versed in aseptic and infection control practices, such as hand hygiene, proper use of disinfectants, and personal protective equipment. The early 1800s lacked any concept of effective antisepsis because they predominantly believed in the miasma theory (now abandoned), which believed that disease was caused by bad air coming out of rotting organic matter. In the era of “miasma theory,” Ignaz Semmelweis dared to pave the way for germ theory disease. Vienna General Hospital supported his work, but his hypothesis remained unpublished and unheard by the rest of the world. In 1861, his major publication, “The etiology, concept, and prophylaxis of childbed fever,” sparked strong opposition and rejection of his theories. His mental condition deteriorated due to the strong rejection and criticism from his peers, leading to the development of amnesia, anxiety, and severe depression. He was unfortunately admitted to an Austrian asylum, where he was confined and beaten. Eventually, the man who conquered puerperal fever succumbed to septicemia due to an infected wound from the beating.

## Introduction and background

In his final days, Ignaz Semmelweis, deeply affected by the criticism he encountered throughout his life, envisioned a world where the occurrence of death caused by preventable infections would come to an end. He articulated "When I look back upon the past, I can only dispel the sadness which falls upon me by gazing into that happy future when the infection will be banished, the destroyer of life annihilated, the weeping mothers will no longer be found, and the number of human beings who die from infection will no longer be counted [1]."

Maternal mortality rates surged during the nineteenth century, leading to a widespread perception of childbirth as a perilous event [2]. People attributed this high mortality to "childbed fever" (puerperal fever), a condition associated with miasma, a mixture of pus, blood, and excrement that polluted the air around the overcrowded wards, now known to be a result of bacterial septicemia. There was no concept of antisepsis or germ theory of disease, and nobody had the grit to challenge the “miasma theory." At the time, bloodletting was the only solution. Amid all the ignorance, Dr. Ignaz Phillip Semmelweis stood as a beacon of truth. His one stroke in introducing hand hygiene practices to Vienna General Hospital was able to save many mother's lives [3]. This article aims to highlight Dr. Ignaz Phillip Semmelweis's immense contribution to infection control, which revolutionized maternal care. His pioneering work in infection control laid the foundation for germ theory and clinical etiological research. Challenging the pre-existing beliefs and norms took a toll on his mental health and life. During his lifetime, he did not receive the recognition he deserved, leading to a life of misery and a tragic death. His life journey exemplifies integrity, perseverance, and humility for the present generation.

## Review

Early life

**Figure 1:**
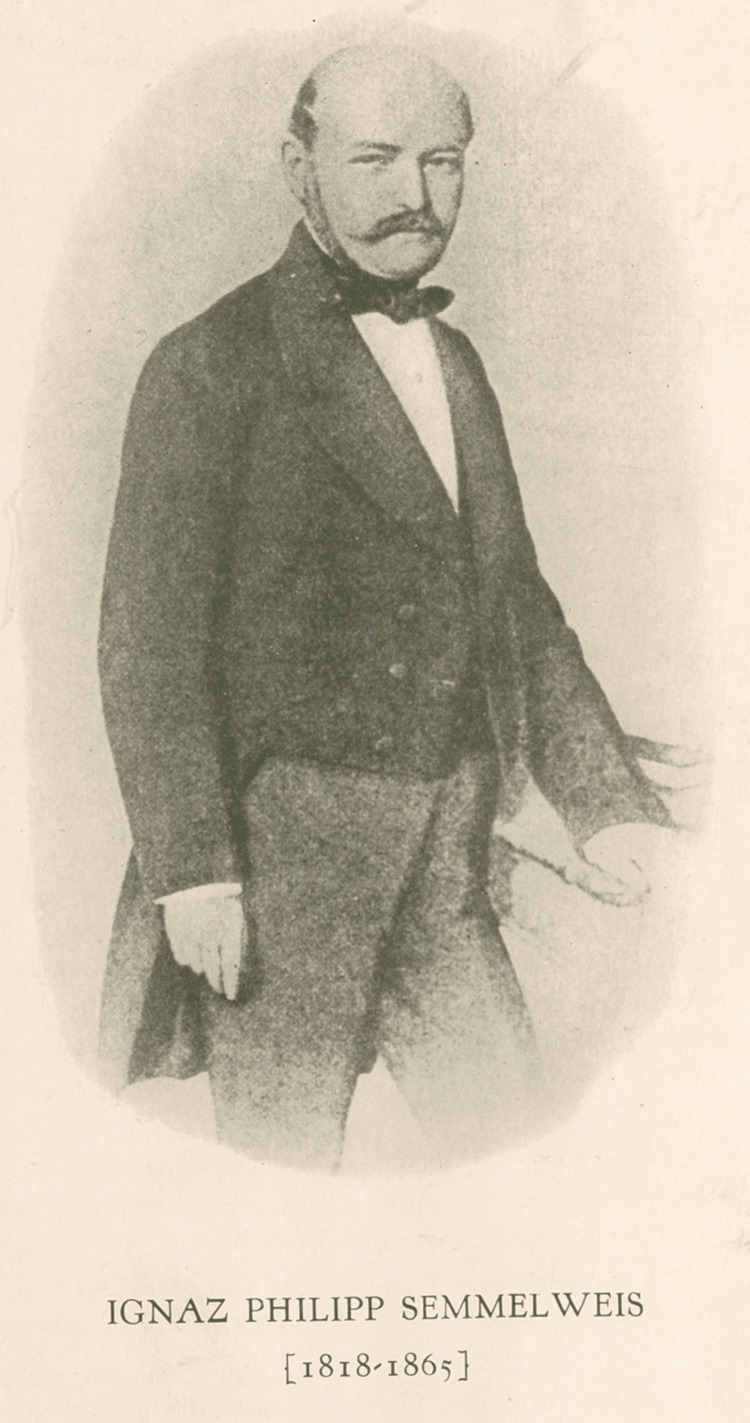
Portrait of Dr. Ignaz Phillip Semmelweiss Source: The image was obtained from Ref. [5] under the Creative Commons Attribution CC BY 4.0 license.

Dr. Ignaz Philip Semmelweis was born on July 1, 1818, in Budapest, Hungary, to a wealthy merchant. He initially enrolled at the University of Vienna to study law but later switched his career to medicine after one year of studying law and graduated as a physician in 1844 [4]. Figure 1 depicts the portrait of Dr. Ignaz Phillip Semmelweis.

Work at Vienna

On July 1, 1846, Professor Johann Klein appointed Dr. Semmelweis as the first assistant obstetrician, an equivalent role to the chief resident, following his unsuccessful attempts to secure a position in internal medicine and pathology [4,6]. There were two divisions of maternity clinics in the Vienna General Hospital. In the first division of the Vienna General Hospital maternity clinic, only medical students attended birth cases, while midwives attended in the second division. Since the division's inception, the first clinic's maternal mortality rate (MMR) was three times higher than the second. Dr. Semmelweis believed the common situations between the two divisions could not have caused this difference. He expanded his research to identify factors that were different between the two divisions and could potentially be the cause. For a considerable amount of time, he was unable to pinpoint the cause of this disparity. However, one day, his friend, Professor Jakob Kolletschka, a forensic pathologist, succumbed to a cut he sustained during an autopsy. Dr. Semmelweis examined Professor Kolletschka's autopsy findings and found them to be identical to those of mothers dying of childbed fever. He concluded that the cause of death in childbed fever is the same as Professor Kolletschka's death. He coined the term “cadaveric particles," which he proposed to have entered Professor Kolletschka's bloodstream; similarly, caregivers introduced them into the mothers' uteri [7].

**Figure 2:**
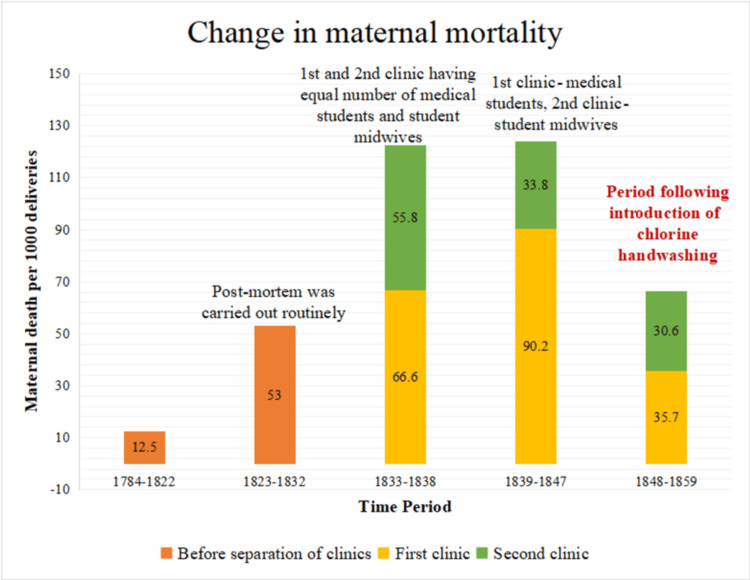
Evolution of maternal deaths per 1000 deliveries in Vienna General Hospital from 1784-1859 Source: The image was created by the authors of this study.

Figure 2 illustrates the evolution of maternal deaths per 1000 deliveries since Vienna General Hospital introduced post-mortem as a routine practice (1823). After the division of maternity clinics in 1833, almost an equal number of medical students and student midwives conducted deliveries in both clinics. However, in 1839, the first division exclusively served medical students, who also performed autopsies, while student midwives conducted deliveries in the second division. It was only after Dr. Semmelweis introduced handwashing practices with chlorine solution in 1848 that the maternal deaths per 1000 deliveries in the first clinic decreased and became almost equal to those in the second clinic [8].

Despite being one of the first to witness the reduction in maternal mortality at Vienna General Hospital, Professor Johann Klein was hesitant to endorse his theory and declined to renew his contract. Frustrated by the non-extension of his contract and the rejection he received, Dr. Semmelweis returned to Hungary and accepted an unpaid position at a St. Rókus Hospital in Pest [9].

Scientific publication

Semmelweis did not publish his findings from Vienna General Hospital, but many young doctors were supportive of them. Soon his results were first published in December 1847 in an editorial of the *Journal of the Society of Physicians *by Ferdinand Hebra, who was then the journal’s editor, thus inviting others to test the findings and report accordingly. However, Semmelweis recognized that his proof faced numerous objections. His seniors and peers opposed him, stating that the reduction in maternal mortality after introducing handwashing was merely a coincidence with the tailing of an epidemic [4].

In 1849, Semmelweis conducted animal studies that were rejected, stating that they were inconclusive and needed more evidence to prove his theory of causality. He could not publish before leaving Vienna or after returning to Pest-Buda, as he was not ready to gather any more evidence to support his theory. After coming to Budapest, his work faced several challenges, like a lack of good academic activity and questioning his theory in the St. Rókus Hospital set-up where no autopsies were carried out [4].

Semmelweis noticed that the MMR had been high at the St. Rókus Hospital since 1847. He found that it was because the chief of surgery, who was also in charge of obstetrics, was spreading the disease from surgical patients to mothers in labor in the same way that it was happening in Vienna: by making rounds on the wards first with surgical patients and then with women who were giving birth, transferring "decaying matter" from wound infections and other purulent discharges [4].

Even though unpaid, Semmelweis joined as Director of the Obstetric Division of St. Rókus Hospital on May 20, 1851, and subsequently reduced the childbed fever with chlorine hand disinfection. However, because of his previous experiences in Vienna, he did not report any of them. After the collection of sufficient cases, his first assistant attempted to publish it in *Vienna Medical Weekly*, which was disregarded, stating it as misleading information [4].

In 1861, Semmelweis published his major work, “Etiology, Concept and Prophylaxis of Childbed Fever.” However, the medical community disapproved of it due to its repetitive nature. When faced with strong opposition, Semmelweis personally responded to each criticism by sending abusive letters to those who disagreed with his views, alienating himself from his peers [10].

Death

The bitter relationship with his medical peers and his inability to prove his hypothesis that saved many lives had a devastating impact on his mental health. It was impossible to determine the cause of his mental disorder; some believed it was due to overwork, stress, and rejection, while others claimed it was third-stage syphilis, a condition common among obstetricians in those eras. Tragic events marked his later life, culminating in his admission to an Austrian asylum by his friend Ferdinand Hebra and his wife Martha. Ignaz Semmelweis succumbed to septicemia within 14 days of admission to the asylum due to a wound infection he contracted from the asylum attendants' beatings. It is ironic how the person who paved the way for antisepsis died of sepsis [11].

Legacy

The "Semmelweis reflex," named after the revolutionary obstetrician Dr. Semmelweis, delineates a critical aspect of human behavior: the inclination to adhere to established norms and resist new ideas that challenge them. This reflex underscores the challenge that individuals frequently encounter when confronted with innovations that contradict established practices. It is imperative to comprehend this concept to fully appreciate Semmelweis's legacy, as it is indicative of his more extensive obstacles in advocating for hand hygiene in obstetrics [12]. The "Semmelweis reflex" is still evident in the medical and scientific communities today. Recent examples of the Semmelweis reflex include the rejection of climate change by various scientific communities and the delay in accepting COVID-19's airborne nature [13]. His story is a testament to the importance of perseverance, critical thinking, and a commitment to improving the human condition, even in the face of adversity. His scientific assessment and prompt intervention led to a decrease in maternal mortality, which gave rise to the science of infectious disease and infection control practices. Not only did he identify the issue, but he was also significantly ahead of his time in providing an immediate solution.

He initiated the germ theory of disease, a concept later advanced by Louis Pasteur (1822-1895), Joseph Lister (1827-1912), and Robert Koch (1843-1910). Ignaz Semmelweis' contribution introduced modern medicine to asepsis, handwashing, and the use of disinfectants, which are the core contributors to infection control practices in the current scenario [14].

Timeline

**Figure 3:**
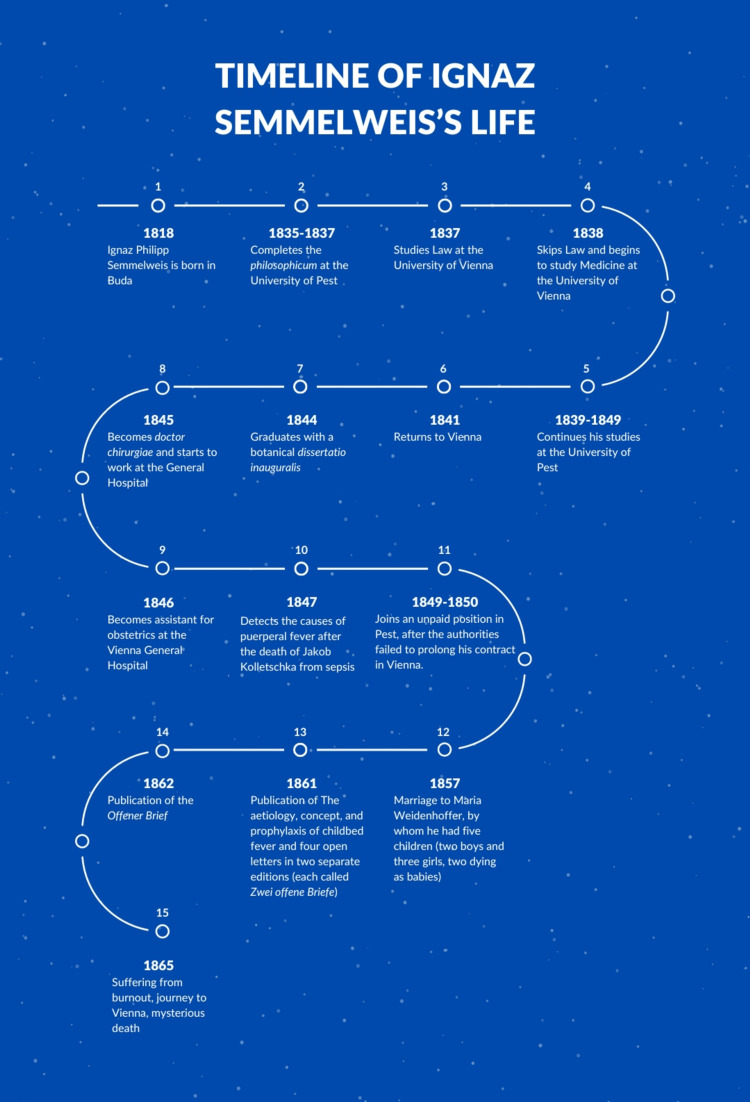
Timeline of Ignaz Semmelweis life Source: The image was created by the authors of this study.

Figure 3 depicts the timeline of important events in the life of Dr. Ignaz Phillip Semmelweis [15].

## Conclusions

Amidst the shadows of darkness, Ignaz Semmelweis was the lone beacon of truth. The truth he stood for ultimately cost him his life. His legacy continues to serve as a testament to the significance of evidence-based medicine and questioning established norms. His life inspires millions of medical researchers across the globe to not only stand for what they believe but also to be humble and open-minded to see what other eyes fail to see and to accept one’s limitations.

His pioneering work in antiseptic procedures has left an indelible mark on the history of healthcare. His relentless pursuit of truth and commitment to the well-being of mothers and infants laid the foundation for modern infection control practices. Today, it is impossible to think of conducting any form of medical intervention without following antiseptic precautions and infection control, a standard we often take for granted. However, there was a time when prominent figures in the medical field not only ignored these ideas but also completely rejected them. Thanks to pioneers like Dr. Ignaz Phillip Semmelweiss, today every hospital operates infection control committees to ensure adherence to antisepsis, which saves countless lives daily. His vision for a world free from preventable infections continues to inspire medical professionals today, underscoring the enduring relevance of his work in our ongoing battle against healthcare-associated infections.
